# Supplementary material for: Can Reduced Intake Associated with Downsizing a High Energy Dense Meal Item be Offset by Increased Vegetable Variety in 3–5-year-old Children?
Source: Nutrients. 2018 Dec 3;10(12):1879. doi: 10.3390/nu10121879 (PMC6315468; doi:10.3390/nu10121879)
Supplement: Supplementary file 1 [file nutrients-10-01879-s001.pdf]

Supplementary Material

Table S1. Ratings for liking of sandwich and vegetables served to children as lunch.

**Food**	**Liking Rating**							
	**Yummy**		**Just Okay**		**Yucky**		**Undecided**	
	n	%	*n*	%	*n*	%	*n*	%
Cheese Sandwich	32	64	6	12	10	20	2	4
Carrot	29	58	6	12	15	30	0	0
Cherry Tomatoes	18	36	3	6	24	48	5	10
Cucumber	25	50	3	6	20	40	2	4
Red Pepper	18	36	3	6	29	58	0	0

Methodology: Food Liking (n=50). The majority of the children (64%) rated the cheese sandwich as 'yummy' (Table S1). Children predominantly rated carrot and cucumber as 'yummy' (58% and 50% respectively) and red pepper and cherry tomatoes as 'yucky' (58% and 48% respectively).
